# Assessing the European Electric-Mobility Transition: Emissions from Electric Vehicle Manufacturing and Use in Relation to the EU Greenhouse Gas Emission Targets

**DOI:** 10.1021/acs.est.2c06304

**Published:** 2022-12-27

**Authors:** Chen Tang, Arnold Tukker, Benjamin Sprecher, José M. Mogollón

**Affiliations:** † Institute of Environmental Sciences, Leiden University, Leiden, 2333 CC, The Netherlands; ‡ Netherlands Organization for Applied Scientific Research (TNO), The Hague, 2595 DA, The Netherlands; § Faculty of Industrial Design Engineering, Delft University of Technology, Delft, 2628 CE, The Netherlands

**Keywords:** climate policy, European e-mobility transition, GHG emission accounting, lithium-ion battery, material flow analysis

## Abstract

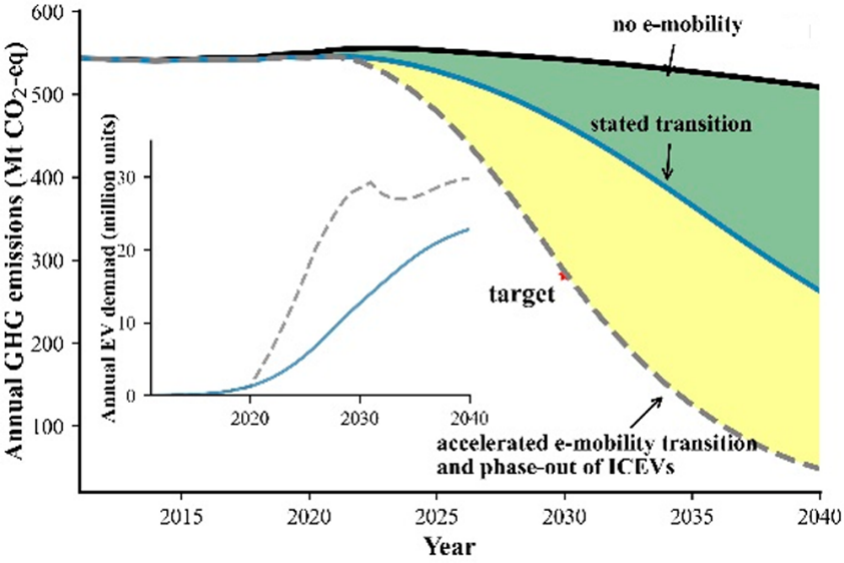

The European Union (EU) has set a 37.5% GHG reduction target in 2030 for the mobility sector, relative to 1990 levels. This requires increasing the share of zero-emission passenger vehicles, mainly in the form of electric vehicles (EVs). This study calculates future GHG emissions related to passenger vehicle manufacturing and use based on stated policy goals of EU Member States for EV promotion. Under these policies, by 2040 the stock of EVs would be about 73 times larger than those of 2020, contributing to a cumulative in-use emission reduction of 2.0 gigatons CO_2_-eq. Nevertheless, this stated EV adoption will not be sufficiently fast to reach the EU’s GHG reduction targets, and some of the GHG environmental burdens may be shifted to the EV battery manufacturing countries. To achieve the 2030 reduction targets, the EU as a whole needs to accelerate the phase-out of internal combustion engine vehicles and transit to e-mobility at the pace of the most ambitious Member States, such that EVs can comprise at least 55% of the EU passenger vehicle fleet in 2030. An accelerated decarbonization of the electricity system will become the most critical prerequisite for minimizing GHG emissions from both EV manufacturing and in-use stages.

## Abstract 2

Meeting the 2030 greenhouse gas reduction target in the EU mobility sector requires accelerating the pace of the e-mobility transition and the rate of decarbonization of the global electricity system.

## 1 Introduction

Passenger vehicle use represents the biggest contributor to greenhouse gas (GHG) emissions from the mobility sector in the European Union (EU), amounting to ca. 545 million tones in CO_2_ equivalent (CO_2_-eq) in 2018.^1^ To address this issue, the EU has set a target to reduce GHG emission to 37.5% of 1990 levels by 2030, mainly via promoting an overall 30% market share of zero-emissions vehicles in new passenger vehicle sales by 2030, with a strong focus on electric vehicles (EVs).^2−4^ EVs come in various forms (e.g., fully battery electric vehicles, BEVs; plug-in hybrid electric vehicles, PHEVs), and together they represent the fastest growing form of zero-emission passenger vehicles.^5^ To align with this near-term EU strategy, Member States have also established their individual climate actions and plans to stimulate the national roll-out of EVs^3^ (e.g., the “Advenir program” in France,^6^ the “Umweltbonus plan” in Germany,^7^ and the “Jedlik Ányos Action Plan” in Hungary,^8^ etc.). These policies differ in ambition with some aiming toward a rapid e-mobility transition (e.g., The Netherlands and Ireland will cease the sales of internal combustion engine vehicles by the end of 2030^2,9^), while others have less ambitious goals (e.g., Germany and France will ban new petrol and diesel vehicle sales with by 2040^2,10^).

Compared to internal combustion engine vehicles (ICEVs), EVs effectively reduce GHG emissions by 30% to 80% when in-use (e.g., while being driven), depending on the energy mix of the electrical grid system.^11−16^ Within the EU, the GHG emissions from in-use EVs have been estimated to decrease by ca. 20–54% between 2015 and 2050 due to an assumed decarbonization of the EU electricity system.^14,17−19^ However, EVs are increasingly powered with a chargeable lithium-ion battery pack (LIBs), which reportedly more than doubles the GHG emissions of manufacturing ICEVs.^15^ This is caused mainly by the energy consumption of extracting and refining raw materials for producing EV batteries. A wide range of estimates of cradle-to-gate GHG emissions of EV battery production can be found in the literature, ranging between 73 and 213 kg CO_2_-eq per kWh of the EV battery energy capacity, determined by the cathode chemistry of EV battery and the carbon intensity of the electricity generation in countries that manufacture EV batteries (e.g., mainly in Asian countries).^20−23^

While previous studies shed light on environmental impacts across the life cycle stages of individual EVs, not many have yet given insight into the annual GHG emissions from driving and producing ICEVs and EVs given the EU Member State ambitions for the e-mobility transition. Whether these stated policies are sufficient to meet the EU carbon targets in 2030 taking into account the projected energy mix of each Member State has yet to be evaluated. Furthermore, there is little information available on the GHG emissions implications for the current EV manufacturing countries.

The aim of this study is to quantify the demand for EVs and the GHG emissions from driving and producing passenger vehicles until 2040, under the stated ambitions of EU countries on the transition toward e-mobility. This analysis combines a dynamic material flow analysis (MFA) for the demand for ICEVs and EVs in combination with GHG emission accounting for passenger vehicle manufacturing and driving. We compare the results with the GHG reduction target set for 2030.

## 2 Data and Methods

The research was conducted in three steps: (1) Classify countries within the study scope and evaluate future demand for passenger vehicles for each country, (2) estimate the future demand for various types of passenger vehicles on the basis of the stated national targets on decarbonization of the mobility sector, and (3) assess annual GHG emissions related to both the in-use and the manufacturing of estimated passenger vehicle fleets. More details for each of the following sections are available in the Supporting Information (SI).

### 2.1 Study Scope

We included 27 EU Member Countries plus the UK, Iceland, and Norway in our study. The 30 countries were separated into two groups, according to the pace of e-mobility transition following each country’s individual ambitions. Detailed targets for each group are listed in Table 1:

**Table 1:** Policy Targets on the Promotion of EVs Sales^10^

		Policy targets on promoting EVs sales	
Group	Countries	2030	2040
High ambition group (HG)	Norway, Austria, Ireland, Iceland, The Netherlands	BEVs sales share 100% of the market by the end of 2030	BEVs sales share 100% of the market
	Belgium, Denmark, France, Germany, Italy, Sweden, Portugal, Finland, Spain, Luxembourg, the U.K.	EVs sales share at least 50% of the market by 2030	EVs sales share 100% of the market by 2040
Low ambition group (LG)	Greece, Hungary, Romania, Lithuania, Poland	EVs sales share at maximum 50% of the market by 2030	Stated promotion targets of EVs sales is not clear beyond 2030; it was assumed to follow the trend to full EVs sales by 2050
	Czech, Croatia, Cyprus, Bulgaria, Estonia, Latvia, Malta, Slovakia, Slovenia	The promotion of EVs sales is not clearly mentioned in the individual climate plans and it was assumed to share 30% of market as same as the overall EU 2030 target^4^	Stated promotion targets of EVs sales is not clear beyond 2030; it was assumed to follow the trend to full EVs sales by 2050

The future of EV mobility was evaluated using two scenarios for the time period 2021–2040. The first “stated transition” scenario assumed the implementation of the policy targets listed in the Table 1. The second “ambitious transition” scenario assumed a faster and ultimately higher rise in market share of EVs, assuming that all the 27 EU + 3 countries follow the policy targets of Norway, Austria, Ireland, Iceland, and The Netherlands. These scenarios were also compared with a baseline scenario, which assumed no additional EVs entering the market from 2021 to 2040 (no e-mobility scenario), to assess the GHG emission savings from the e-mobility transition.

### 2.2 Dynamic MFA Model

In our dynamic market analysis, four types of passenger vehicles were included: BEVs, PHEVs, Hybrid Electric Vehicles (HEVs) and ICEVs. The ICEVs were assumed to be powered by diesel or gasoline. ICEVs powered by natural gas were not considered as a separate group as they have a minimal market share, with their sales accounting for less than 0.6% in 2019 and are mostly concentrated in few countries (e.g., Italy).^24−26^ Fuel Cell Electric Vehicles (FCEVs) were excluded given their minimal 0.04% market share in 2019 and the immature nature of the technology (e.g., high price, insufficient hydrogen fueling stations, limited driving range).^24,27^ We chose the lower-medium size as the average model of the passenger cars, as they have been among the most commonly sold in the European countries.^28^

To quantitatively estimate the demand for passenger vehicles for each country through to 2040, we adapted a dynamic MFA model previously used to assess future material requirements in the Dutch mobility sector.^29−31^ The annual sales and waste for each country *j* in year *t* (Inflow_*(t,j)*_ and Outflow_*(t,j)*_, respectively) were estimated based on in-use stock of passenger vehicles in combination with a vehicle lifespan distribution (*f*(*t*)), as shown in eqs 1–5. As an important driver of flows in the MFA model, the in-use stock of passenger vehicles consisted of the historic stock for each country from 2011 to 2020, collected from Eurostat^32^ and European Automobile Manufacturers Association (ACEA)^33^ (representing the historical model runs), and the prospective stock of passenger vehicles from the year 2021 to 2040 was assumed by a vehicle-to-population ratio and future population growth from the Shared Socioeconomic Pathway, SSP2^34^ (representing the scenario model runs, shown in Figure S1A).

Passenger vehicle lifespan was assumed to follow a Weibull distribution function with scale and shape parameters (λ and *k*, respectively), as shown in eq 5. The average vehicle lifespan for each country was assumed based on a previous study,^35^ based on country-specific historical turnover frequency of passenger vehicles (Table S3). For the analysis in the scenario years (from 2021 onward), the average lifespan of ICEVs was assumed to follow historical trajectories across countries, and the average lifespan of EVs was assumed to be 12 years as suggested by EV automakers^36^ in the no e-mobility scenario and the stated transition scenario. In the ambitious transition scenario, with EVs rapidly dominating the sales market, the average lifespan was assumed to be 12 years for all vehicle types, which assumed an accelerated phase-out of ICEVs to a lower lifespan of 12 years. More details on the assumptions related to lifespan are described in the SI.1.
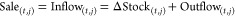
1
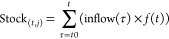
2
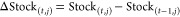
3
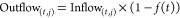
4
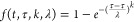
5

The demand for various type of passenger vehicles (*h*) from year 2011 to 2040 for the 27 EU + 3 countries (*D*_*(t,j,h)*_) was estimated on the basis of assumptions for the market share of various passenger vehicles for each country (MS_*(t,j,h)*_), as below:
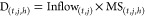
6

The historical market share of various passenger vehicle types was calculated based on the annual numbers of registered passenger vehicles for recent years (2011–2020) collected from the ACEA.^33^ Assumptions for the market share of EVs (BEVs and PHEVs) from 2021 for each country were fitted by the individual future policy targets (Table 1). The future market share of two types of ICEVs (petrol and diesel) and HEVs was assumed to follow the historical trend (Figure S1B). For the scenario with more ambitious e-mobility transition, BEVs would fully dominate the market of passenger vehicles by 2030 within all the 27 EU + 3 countries.

### 2.3 Assessment of GHG Emissions

The GHG emissions associated with the production and use-phase of passenger vehicles in the mobility sector were calculated for each year in the period of 2011 to 2040 (with scenario years from 2021 to 2040), and expressed in carbon dioxide equivalent (CO_2_-eq) mass.^37^

#### 2.3.1 GHG Emissions from Passenger Vehicle Manufacturing

The GHG emissions from the production of passenger vehicles in year *t* were calculated by multiplying the annual demand of various passenger vehicles determined by eqs 1–6 with their GHG emission factors per unit in year *t* (PF_*(t,h)*_) as follows:
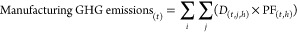
7

The GHG emission factors per unit of various passenger vehicles (PF_*(t, h)*_) were taken from previous studies and were applied to historical years (from 2011 to 2020),^15,21,22,38−40^ listed in Table S4. They were assumed to be dynamic for the scenario years (from 2021 to 2040) determined by the allocation of passenger vehicle manufacturing countries and related reduction of GHG emissions from the electricity generation in those countries, with more details described in Tables 2 and S6. The electricity sources (electricity mixes) have a significant impact on the GHG emissions from electricity generation. We therefore took the historical data (the year 2011–2019) of electricity mixes of manufacturing countries from the statistics data offered by IEA,^41^ and further estimated the future estimated energy mixes for electricity generation (for the year 2020 onward) based on the “stated policies scenario” and “sustainable development scenario” from *IEA Energy Outlook 2020*(42) (Figure S3).

**Table 2:** Assumptions of GHG Emission Factors of Passenger Vehicle Manufacturing for Different Scenarios

		assumptions for scenario years (2021–2040)	
Scenario	E-mobility transition plan	Allocation of the manufacturing countries	Electricity mix of manufacturing countries
No e-mobility scenario	No further EVs entering the EU passenger vehicle market since 2021	Follow the same historical import statistics of EU passenger vehicles collected from ACEA,^33^ as listed in Table S6	Follow the “stated policies scenario” from *IEA Energy Outlook 2020*(42)
Stated transition scenario	The 27 EU + 3 countries follow the individually stated plans for e-mobility transition as listed in Table1	For ICEVs and HEVs, the manufacturing countries will remain in the same trend as historical import statistics;^33^ for EVs, an annual production increase of 1% within the EU, while the remaining demand were assumed to be supplied by non-EU countries according to their historical market shares,^53^ as listed in Table S6 in the SI	Follow the “stated policies scenario” from *IEA Energy Outlook 2020*(42)
Ambitious transition scenario	All the 27 EU + 3 countries follow the policy targets of Norway, Austria, Ireland, Iceland, and The Netherlands as listed in Table1		Follow the “Sustainable Development Scenario” from *IEA Energy Outlook 2020*(42)

Moreover, the GHG emission factor of EV production was also determined by the EV battery capacity.^11,21,22,38−40,43−49^ For PHEVs, their average battery capacity was assumed as 12 kWh,^31^ remaining constant through 2040. The average battery capacity of BEVs from 2011 to 2020 were calculated based on the manufacturing reports of the most popular BEV models sold in EU countries (Table S2). The future battery capacity of BEVs (from 2021 to 2040) was estimated to grow to around 80 kWh by assuming an extended driving range of 550 km, as demonstrated by a previous study^45^ (Figure S4). With all the aforementioned assumptions, the manufacturing GHG emission factors per unit of various passenger vehicle for different scenarios were calculated. More details on assessing GHG emissions from the manufacturing process are described in the SI, section SI.2.1.

#### 2.3.2 GHG Emissions from Passenger Vehicle Use

The annual emissions from the passenger vehicles driving on the road were assessed by multiplying the total annual traveled distance (Vehicle Kilometer Traveled, VKT) with the energy consumption of different types of passenger vehicles and with the respective emission factors related to fuel type or electricity use, as follows:
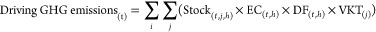
8in which, Stock_*(t,j,h)*_ is various type of passenger vehicles driving on the road for each country calculated by eq 2; EC_*(t,h)*_ is the average energy consumption per traveled distance for various passenger vehicles; DF_*(t,h)*_ is the emission factors per unit energy consumption for various passenger vehicles; VKT_*(j)*_ is the annual traveled distance for each country.

The VKT data of each country was taken from European Environmental Agency,^50^ and assumed to be constant in time in our study (Table S8). The average energy consumption per traveled distance for various passenger vehicles (EC_*(t,h)*_) was individually assumed on the basis of previous studies,^11,38,40,51,52^ with more details described in the SI, section SI.2.2. It is important to note that although the direct driving emissions of GHG of BEVs is zero, the indirect GHG emissions from electricity generation during the charging process obviously need to be accounted for. Therefore, the electricity mixes of each EU country in the scenario years were assumed to follow the IEA scenarios as mentioned above for the scenario analysis in our study (Figure S6).

#### 2.3.3 Uncertainty Analysis

An uncertainty analysis with regard to the future allocation of EV manufacturing countries was performed to assess the influence of carbon-intensity of the electricity grid of production countries on emissions of passenger vehicle production. A Monte Carlo analysis was used to estimate the uncertainty in future GHG emissions of the driven passenger vehicles. The energy consumption of various powertrains was captured in triangular distributions, as the parameter boundaries and most likely values were available to estimate. The variation of annual distance traveled was estimated in normal distribution from the collected data.^33^ More details were described in the SI, section SI.3. A sensitivity analysis was also performed to assess how the GHG emissions would be influenced by the extension of EV and EV battery service time (more details in section SI.5 in the SI).

## 3 Results

### 3.1 Historical Passenger Vehicle Fleet and GHG Emissions

**Figure 1:**
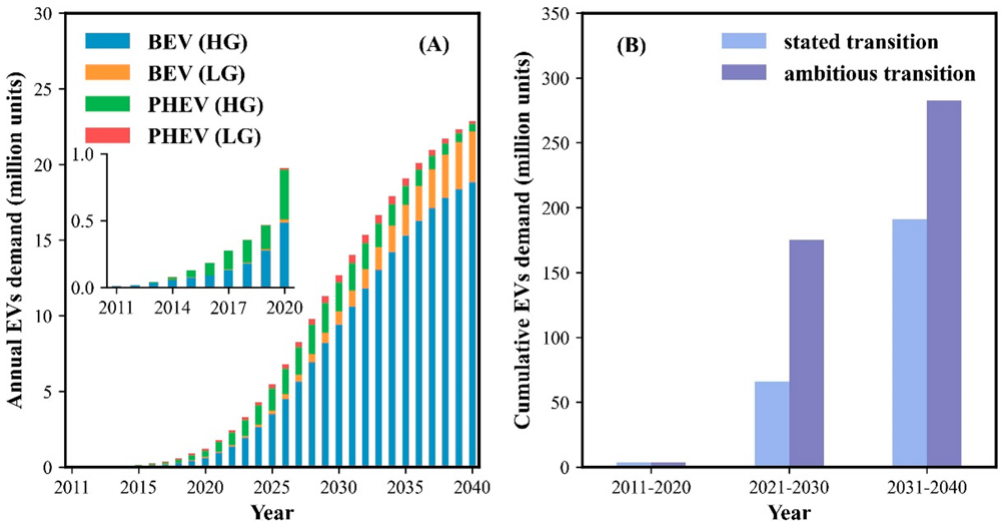
(A) Annual BEVs and PHEVs demand (million units) under the promotion of stated policies through 2040 for countries in the high ambition group (HG) and the low ambition group (LG). Stacked bars in the inserted figure represent historical sales of PHEVs and BEVs by 2020 in the 27 EU + 3 countries. (B) Comparison of cumulative demand for BEVs and PHEVs in the e-mobility transition under the stated transition and the assumption of a more ambitious transition pace.

**Figure 2:**
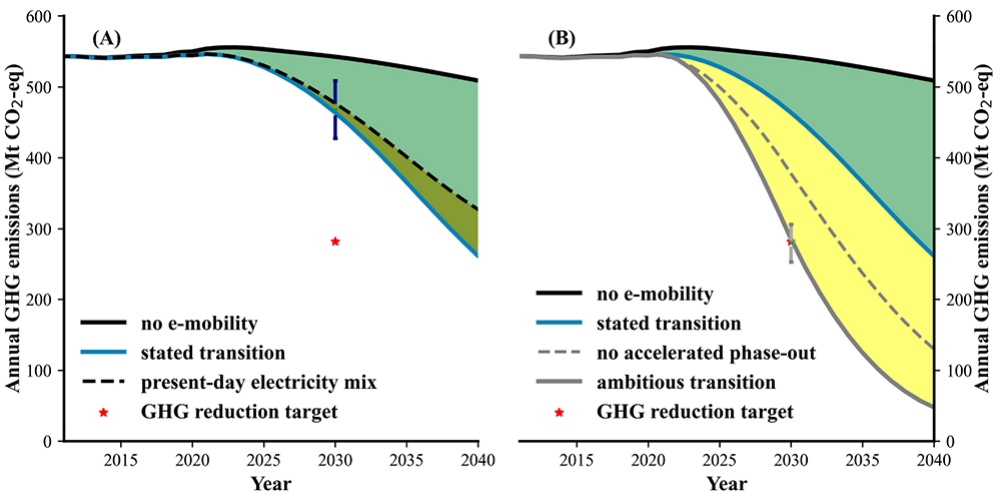
Annual GHG emissions (Mt CO_2_-eq) from the passenger vehicles driving on the road within the 27 EU + 3 countries from 2011 to 2040 under the stated policies on the transition to e-mobility (A) and under the assumption of a more ambitious e-mobility transition (B). The “present-day electricity mix” shown in panel A represents the electricity mix status in the year 2019. The “no accelerated phase-out” shown in panel B represents the GHG emissions performance in the ambitious transition scenario without accelerating phase-out of the ICEVs. The error bar represents the uncertain range of GHG emissions in 2030 and the red star represents the 2030 GHG reduction target set by European Commission on the road mobility sector.^4^

According to EV sale statistics^32,33^ (both BEV and PHEV, see Figure 1 A), EV sales have steadily grown since 2011. With a strong market share increase in 2020, the annual sales of EVs doubled from 2019, reaching to 6% of the overall passenger vehicles market within the 27 EU + 3 countries. By 2020, about 2.5 million units of EVs were sold in total, with 95% of these within high ambition country group. Since 2017, BEVs have a higher market share than PHEVs, comprising 56% of the total EV sales in 2020. Nevertheless, conventional ICEVs still dominate the in-use passenger vehicles. While the fuel use efficiency of ICEV has improved, this has only managed to stabilize GHG emissions at around 540 million tones in CO_2_-eq per year from 2011 to 2020 (Figure 2). Over 80% of these emissions originated from the countries in the high ambition group (Figure S7 in SI).

### 3.2 EV Demand and GHG Emissions from Passenger Vehicle Use

As shown in Figure 2, the annual GHG emissions of passenger vehicle mobility in the 27 EU + 3 countries would only decrease by 9.3% until 2040 compared to the 2020 emission levels without any additional EVs entering the passenger fleet. This improvement is due to the forecasted developments in improved fuel efficiency of ICEVs, and is insignificant compared to the emission reductions that can be expected from an ambitious introduction of BEVs into the market.

In the stated transition scenario, the annual demand for EVs in the coming decades will reach 22.8 million units in 2040, ca. 20 times larger than the 2020 EV sales (Figure 1A). By then, BEVs will account for 94% of the total sales market. Following the stated climate actions on passenger vehicle electrification and an increasing share of renewable sources in electricity generation (Figure S6), the total annual GHG emissions from the driven passenger vehicles will decline from 2023 and reach a close to 52% reduction by 2040 relative to the 2020 levels (Figure 2A). By then, the annual CO_2_ emissions will be reduced by 60% for countries in the high ambition group, and by 20% for the low ambition group, compared to 2020 levels. Moreover, the share of annual CO_2_ emissions from the countries in the high ambition group will drop from 83% in 2020 to 70% in 2040 due to their relatively faster transition pace to e-mobility when compared to the low ambition group (Figure S7). In total, the cumulative GHG emissions savings will be about 2.0 gigatons CO_2_-eq, of which 27% of the emission reduction will arise from the shift to more renewable sources in the electricity supply mix for the 27 EU + 3 countries (assumptions from the “stated policies scenario” by the IEA^42^).

Under the ambitious transition scenario, the annual demand for BEVs will surge up to 29.3 million units in 2030 (Figure S8) or a cumulative demand (2021–2030) that is 2.7 times larger than those for stated policies (Figure 1B). With more BEVs in the vehicle fleet, the annual GHG emissions in 2040 from driving of passenger vehicles will be 93% lower than those of 2020. The faster transition speed will contribute to 2.5 times more emissions reduction compared to the stated policy scenario, cumulatively saving 5.0 gigatons CO_2_-eq between 2020 and 2040, with over 79% of the savings concentrated in the 2030s and 33.2% originated from a faster phase-out of the ICEVs (Figure 2B). While much of the data is subject to uncertainty, a Monte Carlo analysis showed a modest ±10.8% uncertainty range of our annual emission calculations.

### 3.3 GHG Emissions from Passenger Vehicle Manufacturing

**Figure 3:**
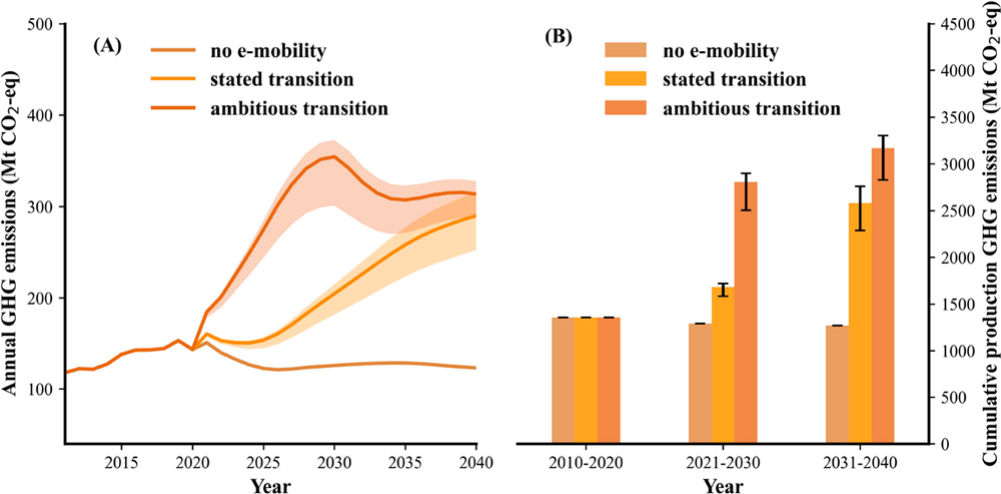
Annual GHG emission (Mt CO_2_-eq) (A) and cumulative GHG emission (Mt CO_2_-eq) (B) from the manufacturing process of the demanded passenger vehicles from 2011 to 2040 during the e-mobility transition under different transition paces. The results bands in (A) represent the range of the aggregated emission level of the electricity system in the manufacturing countries.

**Figure 4:**
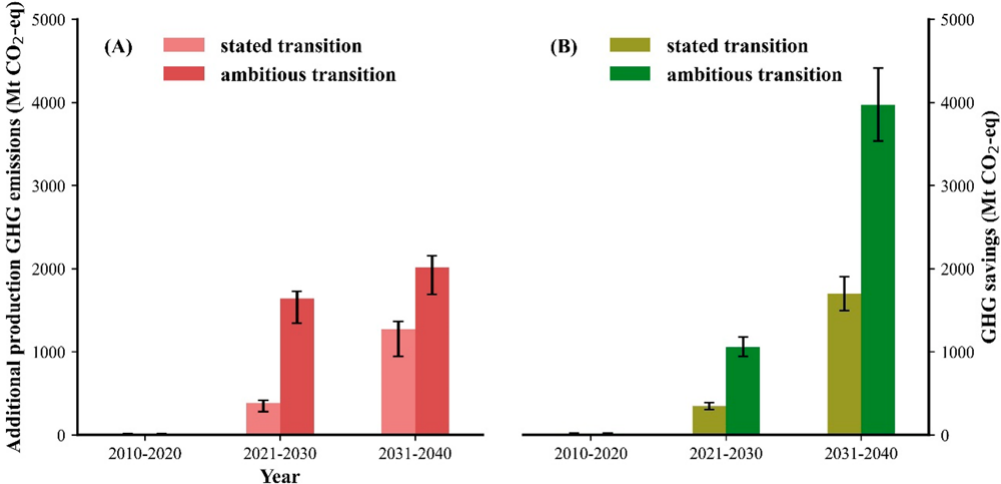
(A) Cumulative additional GHG emissions (Mt CO_2_-eq) from passenger vehicles manufacturing within the 27 EU+ 3 countries under different e-mobility transition paces. (B) Cumulative GHG savings (Mt CO_2_-eq) from the driven passenger vehicles within the 27 EU + 3 countries under different e-mobility transition paces.

In both the stated policy and the ambitious transition scenarios, the market share of BEVs will increase steadily. Since producing EVs is more carbon-intensive than producing ICEVs, the annual GHG emissions during their production process (cradle-to-gate) will also grow. As shown in Figure 3, in the stated transition scenario, by 2040 the annual emissions related to production will be around 2.0 times larger than in 2020, reaching about 290 million tons CO_2_-eq. The cumulative manufacturing emissions since 2021 are projected to reach 4.2 gigatons CO_2_-eq, with over 58% of the total emissions concentrated in the 2030s. The production of EV batteries will become the main emission contributor for passenger vehicle manufacturing during the e-mobility transition, accounting for over 50% of the emissions in the 2030s. Due to the increasing demand for large-sized EV batteries, the cumulative GHG emissions from their production will be at least 31 million tons CO_2_-eq more than the amount of GHG savings from the EVs driven on the road in the 2020s (Figure 4).

Under the ambitious transition scenario, manufacturing emissions will drastically increase caused by the accelerated phase out of ICEVs and hence larger EV demand, reaching around 355 million tons CO_2_-eq in 2030. Emissions will then decline due to the greater decarbonization of the grid system together with a saturation of EV demand post 2030. The higher demand for EVs in the 2020s will lead to a 68% increase in production emissions compared to the stated e-mobility plans, which is also 2.6 times higher than the total GHG reduction from the EVs circulating on the road over the same period. Results further show that by the 2030s, the decadal cumulative GHG emission savings from replacing ICEVs with EVs will begin to offset the cumulative GHG emissions related to producing the required passenger vehicles. The carbon intensity of the electricity mix is also an important source of uncertainty related to the future share of battery production in the various manufacturing countries. With different emission levels and decarbonization rates of the electricity system in the manufacturing countries, the uncertain production allocation leads to about ±5–16% uncertainty range. While not introducing EVs further into the market will lead to a gradual decrease in the following the stated decarbonization of the average electricity system in the manufacturing countries, cumulative carbon savings from adopting a larger EV fleet will be evident already in the 2030s as they will surpass the manufacturing emissions under both scenarios (Figure 4).

Given the optimiztic improvements in EV manufacturing technology, we explored the impact of longer EV lifespans on GHG emissions. Extending the EV lifespan will reduce EV demand from the 2030s, thereby reducing GHG emissions from the manufacturing process. A 53% extension of lifespan for both EV battery and BEV to 18.4 years will contribute to an additional 51% GHG reduction from the manufacturing sector when compared to doubling the BEV usage time with a replaced EV battery, as EV battery manufacturing is very energy intensive (Figure S9).

## 4 Discussion

### 4.1 Meeting the 2030 GHG Reduction Target in the EU Mobility Sector

Given the relatively long remaining lifetime of the existing stock of ICEVs in 2020, the stated pace of the EU e-mobility transition is not sufficient to meet the near-term EU GHG emission reduction target for the mobility sector, which has been set to at least 37.5% GHG emissions reduction by 2030, compared to the 1990 levels.^4^ Assuming equivalent in-use contributions of all mobility sectors, the emission reduction realized by passenger vehicles should be about 188 million tons CO_2_-eq in 2030. Under the current policies of e-mobility promotion and cleaner electricity generation, our results show that the GHG reduction from passenger vehicle use phase in 2030 would be no more than 80 million tons CO_2_-eq. Countries with stronger policy plans for stimulating EVs will reach 15% more annual GHG reduction than those implementing a more conservative e-mobility policy. By then, combustion vehicles will still account for 78% of passenger vehicles circulating on the road. Hence, as also noted by other studies,^40,54^ even if the annual sales of new vehicles shift heavily toward EVs, current vehicle lifetimes do not allow for a sufficiently rapid increase in the share of EVs driving on the road. A crucial conclusion of our modeling is that to meet the goals for 2030 successfully, it is essential to accelerate the replacement of ICEVs by taking them off the road well before their technical end of life, and promoting the uptake of EVs in the 2020s, with BEVs and PHEVs reaching at least 55% of the overall EU fleet in 2030. Furthermore, a phasing out of ICEVs must be accompanied by a total moratorium on new ICEV sales throughout the EU by 2030.

### 4.2 Decarbonizing Global Electric Power Generation

The annual emissions from producing the passenger vehicles needed for the stated EU e-mobility transition will keep increasing until 2040, with the production of EV batteries being the main emission contributor. This result is in line with previous studies.^22,38,46^ The fast development of the EVs sector will thus in part transfer the GHG emission burden from the use of vehicles (e.g., the transport sector) to the production of vehicles (e.g., the industrial sector). This also implies a geographical shift in GHG burdens, as most of the EV manufacturing—and certainly EV battery manufacturing—currently takes place outside the EU countries.^55^ Asian and American manufacturers are dominating the production of EVs and EV batteries because of their mature manufacturing infrastructure.^2^ Although the EU has already announced the establishment of EV battery production facilities in the coming years,^56^ it is unlikely that the EU will be able to internally source most of the EV batteries needed for the e-mobility transition in the near term. Introducing EVs for a low-carbon EU mobility system inevitably shifts the GHG emission burden to manufacturing countries, where the average GHG intensity of electricity is much higher than the EU level.

At the early stage of the e-mobility transition, with most EV batteries produced outside the EU using an electricity system with a relatively high carbon intensity, additional manufacturing GHG emissions will only be partially offset by the saved driving emissions within in the EU countries. Clear benefits of EV implementation will take place in the 2030s, showing that the additional GHG emissions originating from EV battery will be surpassed by the GHG savings from the in-used EVs. The overall benefits will be further enlarged in the ambitious transition scenario, where the greater GHG savings from larger-scale EVs operating on the road due to the accelerated phase-out of the ICEVs will totally offset the overall manufacturing emissions from required passenger vehicles since 2032.

We see hence there are three factors that are crucial for reducing the significant trade-offs of reduced driving emissions (in the EU) with higher production emissions (mainly outside the EU). The first is to accelerate the carbon mitigation of the electricity system simultaneously with the e-mobility transition in the EU countries as well as in EV-producing countries. Greater use of renewable energy to generate electricity will facilitate further reducing GHG emissions by 13% in manufacturing and 11% in the vehicle in-use phase (as reflected in the ambitious scenario). The second is reducing the amount of electrical energy required to produce EV batteries. Electricity consumption was reported to account for over 47.2% of the total energy consumption of EV battery manufacturing. We assumed 120 kWh per battery capacity in this study, in the range of 75–162 kWh of previous studies,^22,47,57,58^ showing an average level of the emissions level based on the present-day technology. Future investment in establishing less energy-intensive technology for EV battery production is necessary to facilitate the EVs going toward lower manufacturing emissions. The third and final is to maximize the environmental benefits from EVs in-use phase, such as extending EV service time, particularly the EV battery use. Longer EV battery lifespans will reduce the demand for EVs and fewer EV battery replacements, contributing to declining the manufacturing emissions. Additional measures that can combine with the promotion of zero-emission passenger vehicles for saving more GHG emissions should also be highly encouraged, such as reducing the average driving energy consumption by promoting light-weighted or smaller-sized passenger cars, optimizing the traveled distances, and reducing vehicle ownership by individuals through, for instance, vehicle sharing schemes.^59^

### 4.3 Study Limitations and Research Outlook

In this study, we have only focused on the GHG performance of e-mobility transition taking EVs as the main decarbonized technology. Future work could include other possible decarbonized technology (e.g., hydrogen based EVs), as well as exploring vehicles for commercial use and public transport for a more complete understanding of the entire mobility sector. Taking the passenger vehicles in lower-medium size as the reference models, the assessment of GHG emissions for the manufacturing process was based on aggregated energy consumption results from previous LCA studies, and for the in-use phase was based on driving patterns influenced by the current usage of passenger vehicles, which were all assumed to remain constant for different scenarios. More detailed modeling for the future studies could include potential improvements in EV battery chemistries and manufacturing process,^60^ changes in driving behavior due to EV adoption (e.g., rebound effects), and reference model changes affected by customer choices to have a more comprehensive investigation of the changes and challenges during the EU e-mobility transition.

## Supporting Information Available

Supporting details on the underlying data and parameters of the material flow analysis, on the assumptions of the emission factors, on the uncertainty analysis, on the additional results of annual driving GHG emissions, and the analysis of the extended lifespan of EVs (PDF)
