# Supplementary material for: Assessing the European Electric-Mobility Transition: Emissions from Electric Vehicle Manufacturing and Use in Relation to the EU Greenhouse Gas Emission Targets
Source: Environ Sci Technol. 2022 Dec 27;57(1):44–52. doi: 10.1021/acs.est.2c06304 (PMC9836351; doi:10.1021/acs.est.2c06304)
Supplement: Supplementary file 1 — es2c06304_si_001.pdf [file es2c06304_si_001.pdf]

Supporting Information

Assessing the European Electric-Mobility Transition: Emissions from Electric Vehicle Manufacturing and Use in Relation to the EU Greenhouse Gas Emission Targets

Chen Tang[1*], Arnold Tukker[1,2], Benjamin Sprecher[3], José M. Mogollón[1]

[1]Institute of Environmental Sciences, Leiden University, Leiden, 2333 CC, The Netherlands

[2]Netherlands Organization for Applied Scientific Research (TNO), The Hague, 2595 DA, The Netherlands

[3]Faculty of Industrial Design Engineering, Delft University of Technology, Delft, 2628 CE, The Netherlands

Supporting information includes: 20 pages, 10 Tables, and 9 Figures.

* Correspondence: Chen Tang (c.tang@cml.leidenuniv.nl)

Contents:

SI.1 Dynamic MFA model S3

SI.2 Assessment of GHG emissions S7

SI.2.1 GHG emissions from passenger vehicle manufacturing S7

SI.2.2 GHG emissions from passenger vehicle use S11

SI.3 Uncertainty analysis S13

SI.4 Additional results S14

SI.5 Lifespan extension S14

Reference: S16

SI.1 Dynamic MFA model

Four types of passenger vehicles were included in this study: Battery Electric Vehicles (BEVs), Plug-in Hybrid Electric Vehicles (PHEVs), Hybrid Electric Vehicles (HEVs), Internal Combustion Engine Vehicles powered by gasoline (ICEV-G) or diesel (ICEV-D). The future stock of passenger vehicles for each country was estimated from historical registration data collected from the Eurostat[1] and the European Automobile Manufacturers Association (ACEA)[2] by assuming a vehicle-to-population ratio and future population growth from the Shared Socio-economic Pathway, SSP2[3] (Figure S1 (A)). The SSP2 scenario outlines a middle-of-the-road scenario in terms of socioeconomic development. It represents moderate population growth and a path in which "social, economic, and technological trends do not shift markedly from historical patterns"[3]. The projected population data was collected from the SSP database[4], and presented at the 2-year intervals in Table S1.

The market share of various passenger vehicle types was calculated based on the annual numbers of registered passenger vehicles for recent years (2011-2020) collected from the ACEA[2]. The assumptions from 2021 for each country were fitted by the individual future policy targets of EVs (BEVs and PHEVs) and the same historical sales trend of HEVs and ICEVs, as shown in Figure S1 (B). The market share of ICEV-P was assumed to be double that of ICEV-D following the historical sales statistics[2]. For the scenario with a more ambitious e-mobility transition, BEVs would fully dominate the market of passenger vehicles by 2030 within all the 27 EU + 3 countries. Best-selling EV models within the 27 EU + 3 countries by 2020 were listed in Table S2.

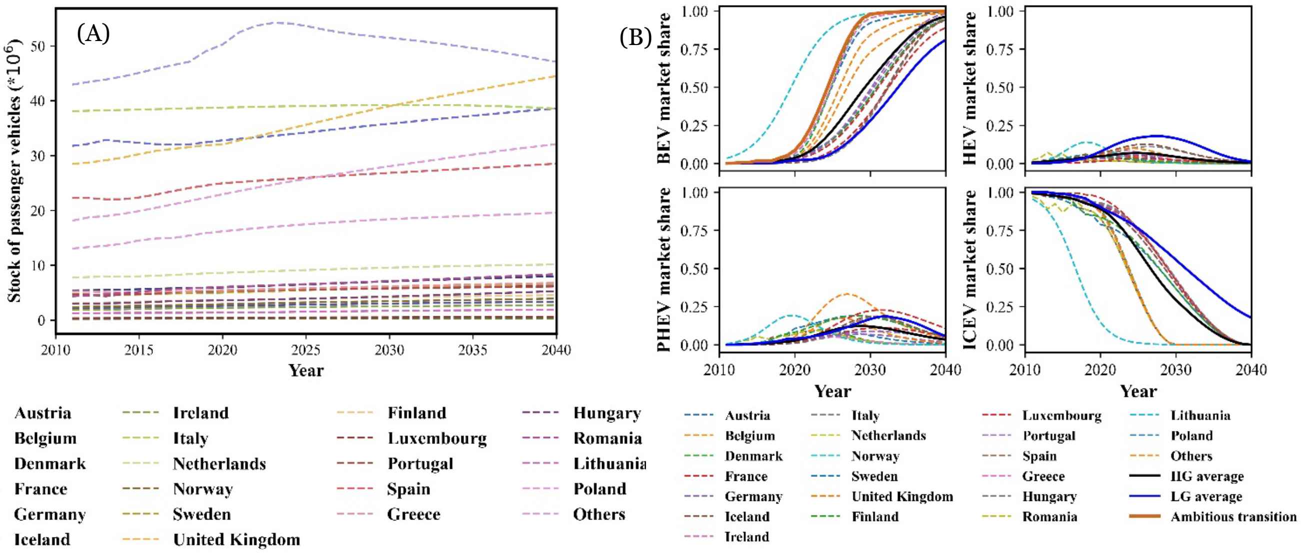

Figure S1. (A) The estimated total stock of passenger vehicles for the 27 EU + 3 countries through 2040. (B) Market share for BEVs, HEVs, PHEVs, and ICEVs of the 27 EU + 3 countries through 2040 following the individual stated e-mobility plans (dashed lines). Solid lines in black and blue represent the average level of the countries in the high ambition group (HG) and low ambition group (LG) following the stated e-mobility transition. The solid line in brown represents the market share of BEVs for the 27 EU + 3 countries in the ambitious transition scenario. ("Others" represents the countries in the low ambition group with the lowest goals in market share of EVs.)

Table S1. Projected population data in European countries (unit: million)[4]

Year	2020	2022	2024	2026	2028	2030	2032	2034	2036	2038	2040
Austria	8.70	8.75	8.81	8.86	8.90	8.95	8.99	9.02	9.05	9.08	9.11
Belgium	11.27	11.37	11.48	11.58	11.68	11.78	11.87	11.96	12.05	12.14	12.22
Denmark	5.81	5.86	5.92	5.97	6.03	6.09	6.14	6.19	6.24	6.29	6.34
France	69.00	69.77	70.60	71.40	72.18	72.94	73.69	74.43	75.15	75.84	76.52
Germany	81.91	81.82	81.73	81.63	81.50	81.36	81.19	81.01	80.81	80.60	80.37
Iceland	0.36	0.36	0.37	0.37	0.38	0.38	0.39	0.39	0.39	0.39	0.40
Ireland	5.04	5.14	5.24	5.33	5.42	5.51	5.60	5.69	5.78	5.87	5.96
Italy	61.70	61.76	61.81	61.87	61.91	61.95	61.99	62.02	62.04	62.04	62.02
Netherlands	17.26	17.40	17.53	17.65	17.77	17.88	17.99	18.08	18.17	18.24	18.31
Norway	5.43	5.54	5.66	5.77	5.89	6.00	6.12	6.23	6.33	6.44	6.54
Sweden	10.19	10.35	10.52	10.68	10.84	10.99	11.13	11.27	11.41	11.56	11.71
United Kingdom	66.21	67.01	67.80	68.57	69.32	70.04	70.74	71.41	72.08	72.74	73.40
Finland	5.62	5.67	5.71	5.76	5.80	5.85	5.88	5.92	5.95	5.98	6.01
Luxembourg	0.59	0.61	0.63	0.64	0.66	0.68	0.69	0.71	0.73	0.74	0.76
Portugal	10.91	10.95	10.98	11.02	11.05	11.08	11.11	11.15	11.18	11.21	11.23
Spain	48.77	49.09	49.39	49.67	49.94	50.21	50.49	50.78	51.07	51.38	51.68
Poland	38.41	38.37	38.31	38.20	38.07	37.89	37.67	37.42	37.16	36.87	36.59
Hungary	9.73	9.68	9.64	9.59	9.54	9.49	9.43	9.38	9.32	9.26	9.20
Romania	20.77	20.61	20.44	20.27	20.09	19.92	19.73	19.54	19.35	19.14	18.94
Lithuania	3.19	3.16	3.14	3.11	3.08	3.05	3.02	2.98	2.95	2.91	2.88
Greece	11.43	11.42	11.41	11.40	11.39	11.38	11.37	11.37	11.36	11.36	11.35
Czech	10.97	11.05	11.12	11.19	11.24	11.29	11.33	11.37	11.40	11.44	11.48
Slovakia	5.61	5.63	5.65	5.66	5.67	5.67	5.66	5.65	5.64	5.63	5.61
Croatia	4.34	4.32	4.31	4.29	4.28	4.26	4.24	4.22	4.20	4.19	4.17
Cyprus	1.25	1.28	1.30	1.33	1.35	1.38	1.40	1.42	1.44	1.46	1.48
Malta	0.43	0.44	0.44	0.44	0.44	0.44	0.45	0.45	0.45	0.45	0.45
Bulgaria	7.07	7.00	6.93	6.87	6.81	6.75	6.70	6.65	6.60	6.55	6.51
Estonia	1.33	1.32	1.31	1.31	1.30	1.30	1.29	1.29	1.28	1.28	1.27
Latvia	2.12	2.10	2.08	2.06	2.04	2.02	2.00	1.98	1.96	1.94	1.93
Slovenia	2.09	2.10	2.11	2.12	2.13	2.14	2.14	2.15	2.15	2.16	2.17

The lifespan of passenger vehicles determines their survival time in the dynamic MFA model. The lifespan was assumed to follow a Weibull distribution function with scale and shape parameters (λ and k), as shown below:

$$f\ (T,\tau,k,\lambda\ ) = 1 -\ e^{-\left(\frac{T-\tau}{\lambda}\right)^{k}}$$

The average lifespan of the passenger vehicles for each country was based on a previous study[5], representing the historical turnover frequency of passenger vehicles. Overall, the average lifespan of the counties in the high ambition group was about 18.4 years, whereas for countries in the low ambition group it was about 24.8 years. The scale and shape parameters (λ and k) are listed in Table S3.

However, the average lifespan of EVs is about 12 years as suggested by many EV automakers[6], representing a survival probability of about 50% after 10.2 years (Figure S2 (A)). Considering the mismatch of lifespans between the conventional ICEVs and the EVs, and we made different assumptions on the lifespans in different scenarios for the assessment in the scenario years (from 2021 onwards). In the no e-mobility scenario and stated transition scenario, the average lifespan of ICEVs in each country was assumed to follow the historical values, and the average lifespan of EVs was assumed to be 12 years. In the more ambitious, however, with EVs rapidly dominating the sales market, the average lifespan was assumed to be 12 years for all vehicle types, which accounts for an accelerated phase-out of ICEVs to a lower lifespan of 12 years. However, for the specific case of Luxemburg the average lifespan for all passenger vehicles in all scenarios was assumed to follow their historical turnover frequency as 8 years. The annual demand for all types of passenger vehicles in different scenarios is shown in Figure S2 (B).

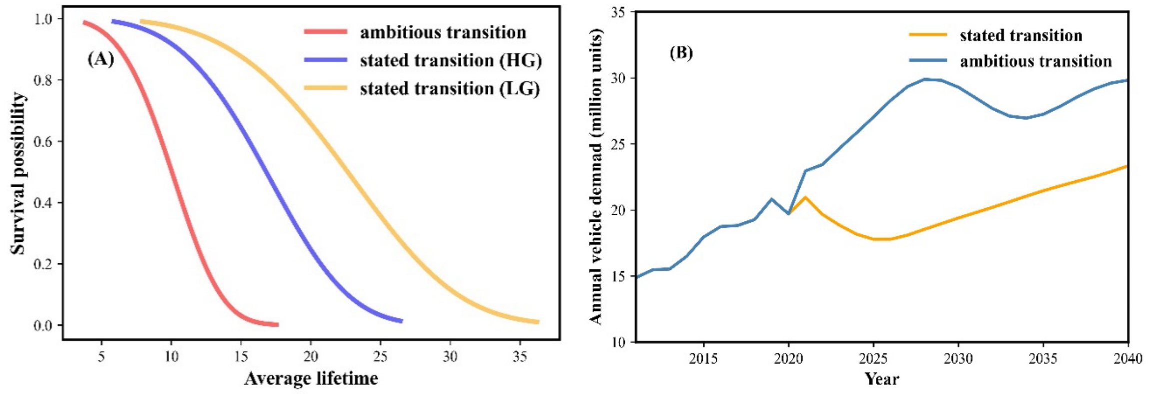

Figure S2. (A) Survival possibility distribution for the average lifespan of passenger vehicles in the stated transition scenario and in the ambitious transition scenario. (B) Annual demand for all types of passenger vehicles in the stated transition scenario and in the ambitious transition scenario.

Table S2. Top sale EV models in European countries by 2020

Vehicle type	EV model	Launched time	Total sales	Battery cathode chemistry	Battery capacity (kWh)	Stated driving range (km)
BEV	Mitsubishi i-MiEV	2010	8119	LMO-poly	16	85
	Peugeot i-On	2010	13823	LMO-poly	14.5	85
	Citroën C-Zero	2010	14314	LMO-poly	14.5	85
	Nissan LEAF (2011)	2011	81811	LMO-poly	24/30	125
	Renault Zoe (2012)	2012	89389	LMO-poly	26	140
	BMW i3 (2013)	2013	95548	LMO-poly	22	130
	Tesla Model S	2013	78541	NCA	75-100	330-490
	SMART-for Two/Four	2013	54022	NMC-111	17	101
	KIA soul	2014	30887	LMO-poly	32	170
	Volkswagen e-Golf	2015	117475	NMC-111	24	130
	Tesla Model X	2016	39978	NCA	60-100	330-490
	Hyundai ioniq	2016	36237	NMC-622	28	190
	Tesla Model 3	2017	181147	NCA	85-100	350-500
	Jaguar I-pace	2017	31970	NMC-622	90	415
	Renault Zoe (2017)	2017	212657	NMC-622	41	255
	Nissan LEAF (2018)	2018	101709	NMC-622	40	245
	Hyundai Kona	2018	73904	NMC-622	39.2	246
	AUDI e-Tron	2018	54022	NMC-622	71.2	330
	Volkswagen ID.3	2020	54495	NMC-622	45	275
	Peugeot e-208	2020	31287	NMC-622	45	275
PHEV	Mitsubishi Outlander	2013	185458	LMO/NMC	12	--
	Volvo V60 Plug-in	2013	41693	NMC-111	10.4	--
	Volkswagen Golf	2014	58271	NMC-111	8.8	--
	Volkswagen Passat	2014	69189	NMC-111	10	--
	AUDI Q5	2019	21099	NMC-622	14.1	--
	Ford Kuga	2020	22628	NMC-622	14.4	--

Table S3. Historical average lifespan of passenger vehicles for each country[5]

Country	Lifespan (λ, k)	Country	Lifespan (λ, k)	Country	Lifespan (λ, k)
Austria	15.9, 3.4	Sweden	19.4, 4.9	Greece	33.9, 4.2
Belgium	11.7, 2.0	United Kingdom	14.2, 4.0	Czech	15.4, 3.6
Denmark	16.9, 3.4	Finland	24.9, 3.2	Slovakia	24.8, 4.03
France	15.2, 6.0	Luxembourg	8.0, 2.0	Croatia	30.9, 6.0
Germany	14.8, 2.4	Portugal	23.1, 6.0	Cyprus	24.8, 4.03
Iceland	19.7, 4.3	Spain	19.4, 3.2	Malta	24.8, 4.03
Ireland	15.0, 4.3	Hungary	23.1, 6.0	Bulgaria	24.8, 4.03
Italy	19.6, 2.7	Romania	24.8, 4.03	Estonia	24.8, 4.03
Norway	19.8, 6.0	Lithuania	24.8, 4.03	Latvia	24.8, 4.03
Netherlands	17.2, 4.4	Poland	24.8, 4.03	Slovenia	20.0, 6.0

SI.2 Assessment of GHG emissions

SI.2.1 GHG emissions from passenger vehicle manufacturing

The GHG emissions from the production of passenger vehicles were calculated by multiplying the total annual demand of various passenger vehicles with their GHG emission factors per unit. We chose the lower-medium size as the average model of the passenger cars, as they have been among the most commonly sold in European countries in recent years[7]. The description of the passenger vehicles powered by different fuel types was listed in Table S4. The production GHG emission factors per manufactured unit for the assessment in the historical years (from 2011 to 2020) were based on the previous studies[8–14]. They were adjusted to correspond to the reference models of the passenger vehicles involved in our study, as listed in Table S4.

Table S4. Passenger vehicle description and historical GHG emission factors for manufacturing process [8–14]

Passenger vehicle type	Description	Main components of the passenger vehicles	GHG emission factors in 2011
ICEV-G	ICEV-G refers to the current most common vehicle technology that burns gasoline to power an engine, with an average weight of 1280 kg per unit.	General glider, powertrain, wheels, transmission, others (e.g. PbA batteries, fluids).	7948 kg/ unit
ICEV-D	ICEV-D refers to a vehicle that burns diesel to power an engine, with an average weight of 1280 kg per unit.	General glider, powertrain, wheels, transmission, others (e.g. PbA batteries, fluids).	7948 kg/ unit
BEV	BEV is powered solely by an electric motor drawing a rechargeable EV battery pack, with an average weight of 1320 kg per unit (including a 22 kWh EV battery pack).	General glider, electric motor and controller, wheels, EV battery pack, others (e.g. fluids).	10167 kg / unit
PHEV	PHEV is a hybrid electric vehicle that has the capability to charge the battery from an off-vehicle electric source, with an average weight of 1400 kg per unit. The probability of the user operating PHEVs in electric mode is set as 0.5.	General glider, powertrain, electric motor and controller, transmission, wheels, EV battery, others (e.g. PbA batteries, fluids).	10324 kg /unit
HEV	HEV draws propulsion energy from both an internal combustion engine or heat engine using consumable fuel, and a piece of rechargeable battery pack getting energy solely from sources onboard the vehicle, with an average weight of 1340 kg per unit.	General glider, powertrain, electric motor and controller, transmission, wheels, EV battery, others (e.g. PbA batteries, fluids).	9447 kg / unit

For the assessment in the scenario years, the use of historical GHG intensity data as aforementioned would create a bias since EV manufacturing has been dominated by countries outside the EU (e.g. China, Japan, Korea and the US)[15]. It is however likely that in future EV production in the EU will catch up. Moreover, the GHG emissions factor of BEVs was also determined by the EV battery capacity[9–13,16–23]. Therefore, we assumed dynamic manufacturing GHG emission factors for the scenario years related to the change of electricity consumption (from 2021 to 2040), determined by the allocation of passenger vehicle manufacturing countries, related reduction of GHG emissions from the electricity generation in those countries, and the dynamic change of average EV battery capacity for EVs. The average electricity consumption for various types of passenger vehicles and EV battery manufacturing are listed in Table S5. The GHG emissions related to other forms of energy consumption were assumed to remain constant in time for all passenger vehicles and the EV battery pack (listed in Table S5) [18–21,24–28].

Table S5. Average electricity consumption for passenger vehicle manufacturing

Vehicle type and EV battery	Electricity consumption from manufacturing process	GHG emission factor from other forms of energy consumption	References
ICEV-G	7257 kWh / unit	4882 kg/ unit	Moreno[14]; Hawkins et al. [20]
ICEV-D	7257 kWh / unit	4882 kg/ unit	Moreno[14]; Hawkins et al. [20]
BEV (without EV battery)	6580 kWh / unit	4562 kg /unit	Moreno[14]; Hawkins et al. [20]
PHEV (without EV battery)	8424 kWh / unit	5353 kg/unit	Moreno[14]; Milovanoff et al.[24] ; Majeau-Bettez et al. [25]; Onat et al.[28]
HEV (without EV battery)	8239 kWh / unit	5277 kg/unit	Moreno[14] ; Milovanoff et al.[24]
EV battery pack	120 kWh / kWh battery capacity	47.2 kg/ kWh battery capacity	Moreno[14]; Sun et al.[21]; Dai et al.[26], Ellingsen et al.[18,27]

The distribution of manufacturing countries for different scenarios was assumed based on the EU historical import statistics of various passenger vehicles in the 2010s collected from ACEA[2]. For the no e-mobility scenario, the prospective allocation of manufacturing countries was assumed to remain constant until 2040. For the scenarios in which EVs promotion would take place (stated transition scenario and ambitious transition scenario), we assumed an annual increase of 1% in EV production within the EU, while the remaining demand for EVs was assumed to be supplied by non-EU countries according to their historical manufacture market shares[29], as listed in Table S6. The manufacturing allocation of ICEVs and HEVs in these two scenarios was assumed to keep the same trend as in the historical years.

Table S6. Allocation of the manufacturing countries for various passenger vehicles in different scenarios

Vehicle type	Historical allocation of the manufacturing countries (from 2011 to 2020)[2,29]	Prospective allocation of the manufacturing countries (from 2021 to 2040)
ICEVs and HEVs	27 EU + 3 countries (65%), Japan (10%), Turkey (8%), Korea (6%), The U.S. (6%), Others (5%)	No e-mobility scenario: same trend as historical years.<br>Scenarios with EV promotion: same trend as historical years.
PHEVs and BEVs	China (39.5%), Korea (32%), Japan (15%), The U.S. (13.5%)	No e-mobility scenario: same trend as historical years.<br>Scenarios with EV promotion: An annual increase of 1% in manufacturing share within the EU, with non-EU manufacturing keeping the historical market share of exports to the EU.

The choice of electricity sources (electricity mixes) has a significant impact on the GHG emissions from electricity generation. In this study, we therefore took the historical data (the year 2011-2019) of electricity mixes of manufacturing countries from the statistics data offered by IEA[30] and the estimated energy mixes (from the year 2020 onwards) based on the "stated policies scenario" and "sustainable development scenario" from IEA Energy Outlook 2020[31]. The IEA scenarios included the forecast for the share and compound average annual growth rate (CAAGR) of each resource until 2040. The electricity mixes for the scenarios were assigned based on the contribution share to the future goal of the total electricity production volume from each country (Figure S3). Eight major resources for electricity generation and the carbon equivalent emission factors for each source were taken from the previous study[32] and listed in Table S7.

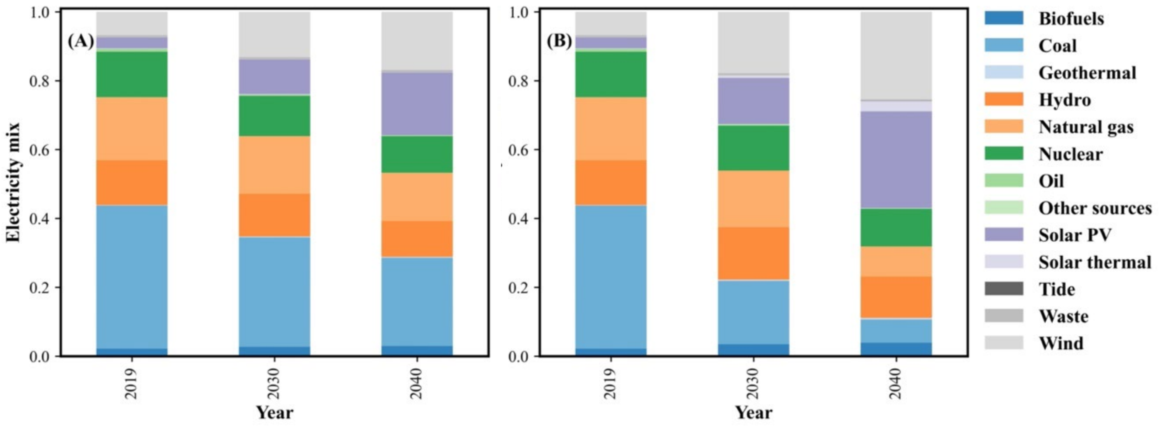

Figure S3. Integrated electricity mix of all passenger vehicle manufacturing countries in 2019, 2030 and 2040, for (A) the stated transition scenario, and (B) the ambitious transition scenario. The allocation of the manufacturing countries was described in Table S6 and the electricity mix in 2030 and 2040 followed the prediction by the "stated policy scenario" and "sustainable development scenario" in the report from IEA[31].

Table S7. GHG emission factors of different types of fuel in electricity generation[32]

Fuel type	Biomass	Coal	Oil	Natural gas	Solid waste	Wind	Solar	Nuclear	Hydropower
GHG emission factors (g CO2-eq/ kWh)	230	820	730	490	52	11	44	12	24

The average battery capacity of BEVs from 2011 to 2020 was calculated based on the manufacturing reports of the most popular BEV models sold in EU countries (Table S2). The future battery capacity of BEVs (from 2021 to 2040) was estimated at around 80 kWh by assuming an extended driving range of 550 km[19], as shown in Figure S4. For PHEVs, their average battery capacity was assumed as 12 kWh[33], remaining constant through 2040.

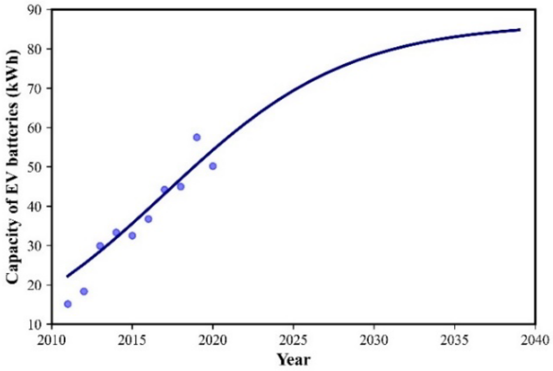

Figure S4. Estimated power capacity of EV battery used in BEVs based on a driving range assumptions of 550 km. The scatters represent average battery power capacity of the lunched BEV models by 2020 as listed in Table S2.

With all the aforementioned assumptions, the manufacturing GHG emission factor for different scenarios were calculated for per unit various passenger vehicle and shown in Figures S5.

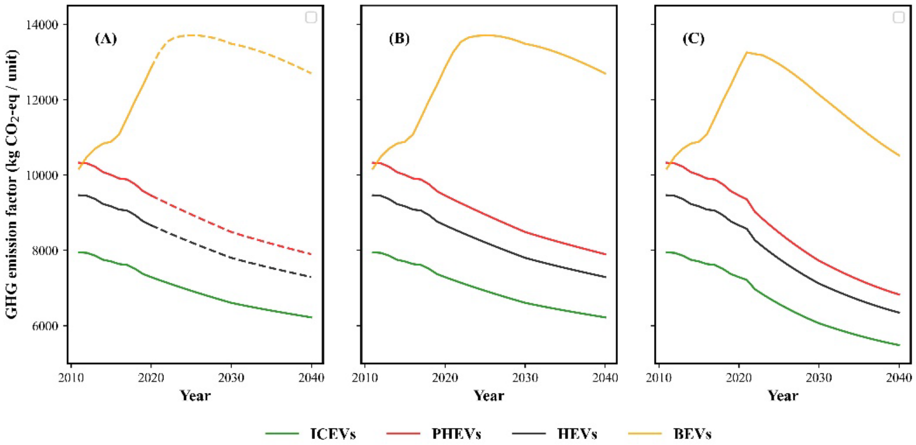

Figure S5. Production GHG emission factors per unit passenger vehicle for (A) no e-mobility scenario, (B) stated transition scenario, and (C) ambitious transition scenario.

SI.2.2 GHG emissions from passenger vehicle use

The annual emissions from the in-use passenger vehicles were assessed by multiplying the total annual traveled distance (Vehicle Kilometer Travel, VKT, listed in Table S8) with the energy consumption of different types of passenger vehicles and with the respective emission factors related to fuel type or electricity use. The assumptions on their average in-use energy consumption from 2010 to 2040 were listed in Table S9.

In our model, we incorporated a decrease in fuel consumption of new ICEVs due to the improved technologies toward 2040. Using the historical fuel consumption values in 2000 as the initial values (7.6 L /100 km for petrol and 6.2 L /100 km for diesel), the fuel consumption remained annually decreased at the rate of 1.14% and 1.34% for petrol and diesel[34]. Emission factors of the passenger vehicles with fuel consumption (ICEVs, HEVs and PHEVs) were 2.31 kg CO_2-eq / L and 2.69 kg CO_2-eq / L for petrol and diesel, taken from a previous study[12].

The fuel consumption of HEVs is 30% – 50% less than that of a comparable ICEVs and was also assumed to remain constant until 2040, as hybrid systems have been taken as a bridge to meeting tougher tailpipe-emissions requirements and the automakers are focusing more on the zero-emission passenger vehicles (e.g., BEVs)[35].

The total energy consumption of PHEVs depends strongly on the driving and charging patterns of vehicle users to choose the driving mode, and it is hard to precisely estimate. Therefore, we set the parameter of this probability as 0.5, which means that half of the total energy consumption per traveled distance contributes from the electricity and the other half contributes from fuel (only gasoline-electricity PHEVs were considered in this study). The energy consumption of PHEVs was taken from the previous study and assumed to be constant until 2040[10].

For the BEVs, the average electric energy consumption from basic driving and charging loss[12], was assumed to be dynamic following the changes in the EV battery capacity as shown in Figure S4. The energy consumption for BEVs was calculated based on an average of 5.4 Wh per additional 100 kilograms in vehicle weight, as demonstrated by a previous study[18]. The weight changes in glider size were negligible compared to the changes in EV battery weight and thus were neglected in this study. Other factors such as motor efficiency, cargo load and driving behavior were not included. The electricity mixes of each EU country in the scenario years were assumed to follow the IEA scenarios[31], as shown in Figure S6.

Table S8. Annual distance travelled (VKT) by the passenger vehicle for each country[36]

Country	VKT (km)	Country	VKT (km)	Country	VKT (km)
Austria	14100	Sweden	12000	Greece	11500
Belgium	14770	United Kingdom	12000	Czech	8000
Denmark	16000	Finland	15000	Slovakia	8000
France	12000	Luxembourg	14000	Croatia	16000
Germany	14700	Portugal	13060	Cyprus	11000
Iceland	10500	Spain	12500	Malta	8000
Ireland	17000	Hungary	13000	Bulgaria	7000
Italy	10500	Romania	10000	Estonia	14000
Norway	15000	Lithuania	12000	Latvia	11000
Netherlands	13200	Poland	8000	Slovenia	8000

Table S9. Passenger vehicle type description and average energy consumption of in-use phase

Vehicle type	Fuel type	Average driving energy consumption	References
ICEV-G	Unleaded petrol E5 (L)	6.8 L / 100 km in 2011<br>6.1 L / 100 km in 2020<br>5.5 L / 100 km in 2030<br>4.9 L /100 km in 2040	Molovanoff et al.[10];<br>Küfeoğlu et al.[12];<br>Uson et al.[34] ;<br>Sihvonen et al. [35];
ICEV-D	Diesel fuel B7 (L)	5.4 L / 100 km in 2011<br>4.7 L / 100 km in 2020<br>4.2 L /100 km in 2030<br>3.7 L / 100 km in 2040	Molovanoff et al.[10] ;<br>Uson et al.[34] ;<br>Sihvonen et al. [35];
BEV	Electricity (kWh)	15.6 kWh / 100 km in 2011<br>16.8 kWh / 100 km in 2020<br>17.6 kWh / 100 km in 2030<br>17.9 kWh / 100 km in 2040	Ellingsen et al.[18];<br>Zhang et al.[37];<br>Cox et al.[38]
PHEV	Only unleaded petrol E5 (L)+<br>Only electricity (kWh)	6.7 L /100km +<br>21.7 kWh /100km	Küfeoğlu et al.[12];<br>Zhang et al.[37]
HEV	Unleaded petrol E5 (L)	4.8 L / 100km	Molovanoff et al.[10]

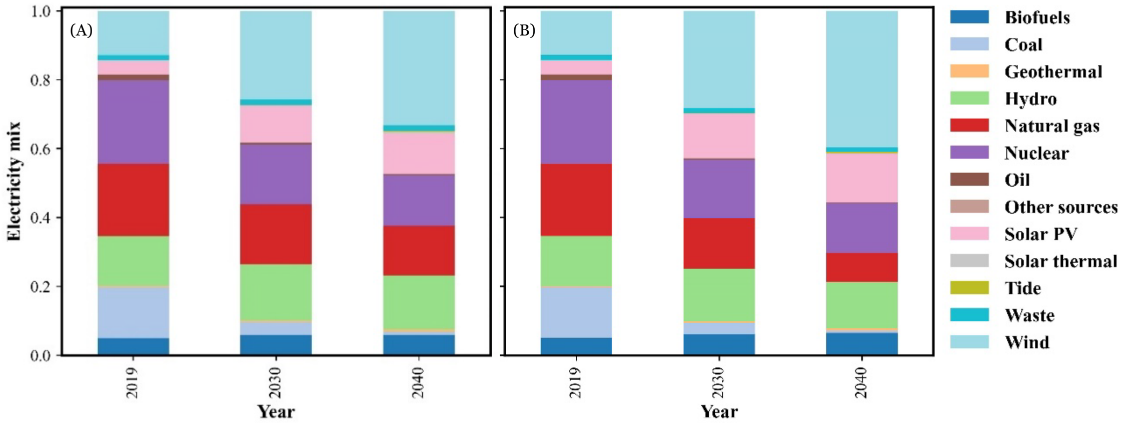

Figure S6. Electricity mix of the 27 EU + 3 countries in 2019, 2030 and 2040. The electricity mix in 2030 and 2040 followed the prediction by the (A) "stated policy scenario" and (B) "sustainable development scenario" in the report from IEA[31].

SI.3 Uncertainty analysis

A Monte Carlo analysis was used to estimate the uncertainty in future GHG emissions of the in-use passenger vehicles from the input parameters in our model. Table S10 lists specific distributions of the related input parameters to model the GHG emissions.

Table S10. Input parameters description of the uncertainty analysis

Input parameter	Unit	Distribution	Base value	Value range (lower, upper)	References
Annual distance travelled	km	Normal	listed in Table S8	±10% of the base value	ACEA[2]
Fuel consumption of ICEVs	L / 100 km	Triangular	dynamic, listed in Table S9	85% to 118% of the base value	Hawkins et al.[8]
Fuel consumption of HEVs	L / 100 km	Triangular	4.8	4.08 - 5.9	Molovanoff et al.[39]
Energy consumption of BEVs	kWh / 100 km	Triangular	dynamic, listed in Table S9	96% to 106% of the base value	Zhang et al.[37]; Ellingsen et al.[18]
Energy consumption of PHEVs	L / 100 km	Triangular	6.7	5.8 - 7.2	Küfeoğlu et al.[12]; Zhang et al.[37]
	kWh / 100 km		0.217	0.2 - 0.223	
Driving model probability of PHEVs	none		0.5	0 - 1	

SI.4 Additional results

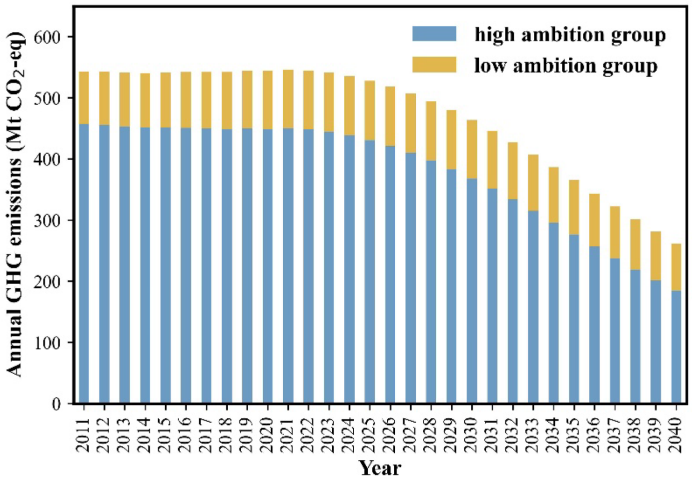

Figure S7. Annual GHG emission (Mt CO_2-eq) from driving passenger vehicles within the 27 EU + 3 countries until 2040 during the e-mobility transition under the stated policies.

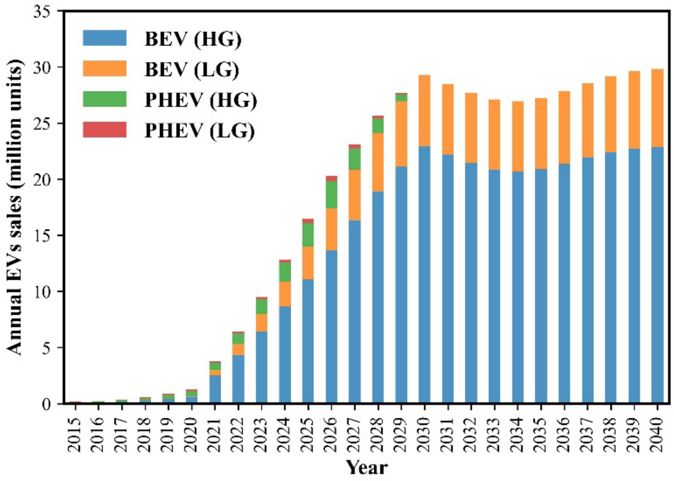

Figure S8. Annual BEVs and PHEVs demand (million units) under the promotion of a more ambitious e-mobility transition pace and an accelerated phase-out of ICEVs for countries in the high ambition group (HG) and the low ambition group (LG).

SI.5 Lifespan extension

A sensitivity analysis was performed for the ambitious transition scenario to assess how the extension of EV lifetime will influence GHG emissions. We explored two options for extending the lifespan of BEV, including:

- An **extended BEV use** from 12 years to 24 years, assuming that the replacement of EV battery would happen when the first EV battery reaches its end of life and the replaced EV battery has a lifespan of 12 years;

- An **extended BEV lifespan** from 12 years to 18.4 years, representing an optimistic assumption that future EV battery technology would improve to the point of enabling BEVs to meet the historical average lifespan of the ICEVs in the high ambition group.

As shown in Figure S9, there are no major differences in GHG emissions from the manufacturing process at the early stage of the e-mobility transition (in the 2020s). Cumulative EV demand in the 2030s can decrease by 34% by expanding EV lifetimes from 12 to 24 years. This demand decrease would lead to a 615 million tons drop in manufacturing GHG emissions. Nonetheless, although an extension of lifespan for both EV batteries and BEV from 12 years to 18.4 years would lead to a 27% decrease in cumulative EV demand, it would lead to 930 million tons of GHG reductions from the manufacturing process, as it reduces the production of energy-intensive EV batteries. Longer EV battery lifespans will allow for longer EV service time and fewer EV battery replacements, contributing to greater improvement in the environmental benefits of BEV adoption.

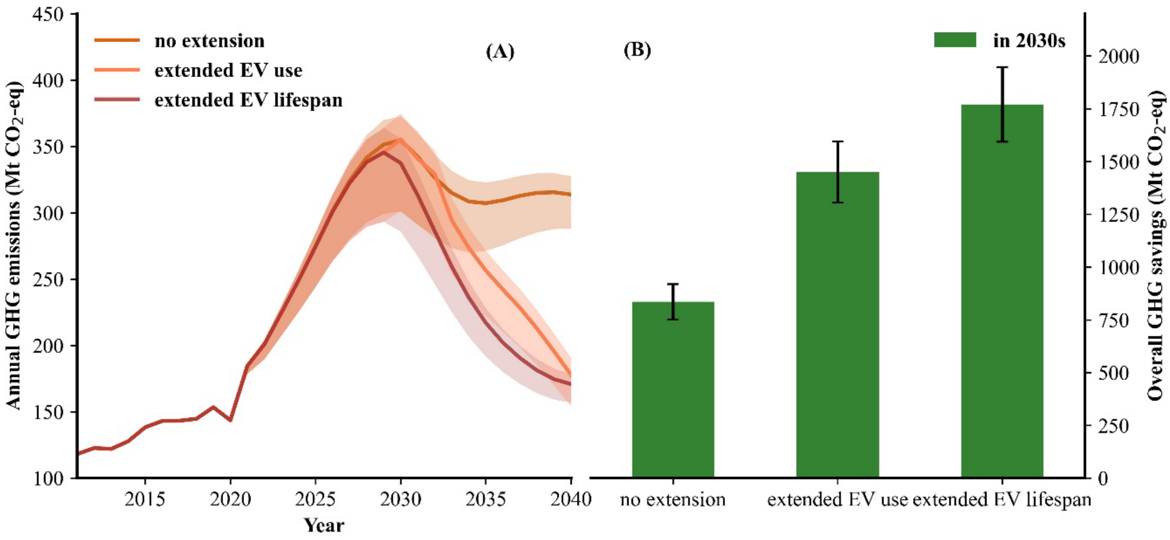

Figure S9. (A) Annual GHG emissions from the production of demanded passenger vehicles following different lifespan extension options under the ambitious transition scenario. (B) Overall GHG savings (difference between driving GHG emission reductions and manufacturing GHG emissions) in the 2030s following different lifespan extension options under the ambitious transition scenario.

Reference:

(1) European Commission. *Database - Eurostat*. 2017. https://ec.europa.eu/eurostat/data/database (accessed 2021-02-24).

(2) *ACEA - European Automobile Manufacturers' Association*. https://www.acea.be/statistics (accessed 2021-02-24).

(3) Riahi, K.; van Vuuren, D. P.; Kriegler, E.; Edmonds, J.; O'Neill, B. C.; Fujimori, S.; Bauer, N.; Calvin, K.; Dellink, R.; Fricko, O.; Lutz, W.; Popp, A.; Cuaresma, J. C.; KC, S.; Leimbach, M.; Jiang, L.; Kram, T.; Rao, S.; Emmerling, J.; Ebi, K.; Hasegawa, T.; Havlik, P.; Humpenöder, F.; da Silva, L. A.; Smith, S.; Stehfest, E.; Bosetti, V.; Eom, J.; Gernaat, D.; Masui, T.; Rogelj, J.; Strefler, J.; Drouet, L.; Krey, V.; Luderer, G.; Harmsen, M.; Takahashi, K.; Baumstark, L.; Doelman, J. C.; Kainuma, M.; Klimont, Z.; Marangoni, G.; Lotze-Campen, H.; Obersteiner, M.; Tabeau, A.; Tavoni, M. The Shared Socioeconomic Pathways and Their Energy, Land Use, and Greenhouse Gas Emissions Implications: An Overview. *Global Environmental Change* **2017**, *42*, 153–168. https://doi.org/10.1016/j.gloenvcha.2016.05.009.

(4) *SSP Database*. https://tntcat.iiasa.ac.at/SspDb/dsd?Action=htmlpage&page=10 (accessed 2020-11-01).

(5) Held, M.; Rosat, N.; Georges, G.; Pengg, H.; Boulouchos, K. Lifespans of Passenger Cars in Europe: Empirical Modelling of Fleet Turnover Dynamics. *European Transport Research Review* **2021**, *13* (1). https://doi.org/10.1186/S12544-020-00464-0.

(6) Harper, G.; Sommerville, R.; Kendrick, E.; Driscoll, L.; Slater, P.; Stolkin, R.; Walton, A.; Christensen, P.; Heidrich, O.; Lambert, S.; Abbott, A.; Ryder, K.; Gaines, L.; Anderson, P. Recycling Lithium-Ion Batteries from Electric Vehicles. *Nature*. 2019, pp 75–86. https://doi.org/10.1038/s41586-019-1682-5.

(7) Campestrini, M.; Mock, P. European Vehicle Market Statistics. *International Council on Clean Transportation* **2021**, 1–50.

(8) Hawkins, T. R.; Singh, B.; Majeau-Bettez, G.; Strømman, A. H. Comparative Environmental Life Cycle Assessment of Conventional and Electric Vehicles. *J Ind Ecol* **2013**, *17* (1), 53–64. https://doi.org/10.1111/j.1530-9290.2012.00532.x.

(9) Kim, H. C.; Wallington, T. J.; Arsenault, R.; Bae, C.; Ahn, S.; Lee, J. Cradle-to-Gate Emissions from a Commercial Electric Vehicle Li-Ion Battery: A Comparative Analysis. *Environ Sci Technol* **2016**, *50* (14), 7715–7722. https://doi.org/10.1021/acs.est.6b00830.

(10) Milovanoff, A.; Kim, H. C.; De Kleine, R.; Wallington, T. J.; Posen, I. D.; MacLean, H. L. A Dynamic Fleet Model of U.S Light-Duty Vehicle Lightweighting and Associated Greenhouse Gas Emissions from 2016 to 2050. *Environ Sci Technol* **2019**, *53* (4), 2199–2208. https://doi.org/10.1021/acs.est.8b04249.

(11) Nealer, R.; Hendrickson, T. P. Review of Recent Lifecycle Assessments of Energy and Greenhouse Gas Emissions for Electric Vehicles. *Current Sustainable/Renewable Energy Reports* **2015**, *2* (3), 66–73. https://doi.org/10.1007/s40518-015-0033-x.

(12) Küfeoğlu, S.; Khah Kok Hong, D. Emissions Performance of Electric Vehicles: A Case Study from the United Kingdom. *Appl Energy* **2020**, *260*, 114241. https://doi.org/10.1016/j.apenergy.2019.114241.

(13) Notter, D. A.; Gauch, M.; Widmer, R.; Wäger, P.; Stamp, A.; Zah, R.; Althaus, H. J. Contribution of Li-Ion Batteries to the Environmental Impact of Electric Vehicles. *Environ Sci Technol* **2010**, *44* (17), 6550–6556. https://doi.org/10.1021/es903729a.

(14) Moreno Ruiz E., F. D. , S. A. , I. D. ,Müller J. , V. L. , V. C. , M. N. , S. T. Documentation of Changes Implemented in the Ecoinvent Database v3.8. **2021**.

(15) *Top electric vehicle markets dominate lithium-ion battery capacity growth | S&P Global Market Intelligence*. https://www.spglobal.com/marketintelligence/en/news-insights/blog/top-electric-vehicle-markets-dominate-lithium-ion-battery-capacity-growth (accessed 2021-04-01).

(16) Delogu, M.; Zanchi, L.; Dattilo, C. A.; Pierini, M. Innovative Composites and Hybrid Materials for Electric Vehicles Lightweight Design in a Sustainability Perspective. *Mater Today Commun* **2017**, *13*, 192–209. https://doi.org/10.1016/j.mtcomm.2017.09.012.

(17) Li, J.; Yang, B. Analysis of Greenhouse Gas Emissions from Electric Vehicle Considering Electric Energy Structure, Climate and Power Economy of Ev: A China Case. *Atmos Pollut Res* **2020**, *11* (6), 1–11. https://doi.org/10.1016/j.apr.2020.02.019.

(18) Ellingsen, L. A.-W.; Singh, B.; Strømman, A. H. The Size and Range Effect: Lifecycle Greenhouse Gas Emissions of Electric Vehicles. *Environmental Research Letters* **2016**, *11* (5), 054010. https://doi.org/10.1088/1748-9326/11/5/054010.

(19) Notter, D. A.; Gauch, M.; Widmer, R.; Wäger, P.; Stamp, A.; Zah, R.; Althaus, H.-J. Contribution of Li-Ion Batteries to the Environmental Impact of Electric Vehicles. *Environ Sci Technol* **2010**, *44* (17), 6550–6556. https://doi.org/10.1021/es903729a.

(20) Hawkins, T. R.; Singh, B.; Majeau-Bettez, G.; Strømman, A. H. Comparative Environmental Life Cycle Assessment of Conventional and Electric Vehicles. *J Ind Ecol* **2013**, *17* (1), 53–64. https://doi.org/10.1111/j.1530-9290.2012.00532.x.

(21) Sun, X.; Luo, X.; Zhang, Z.; Meng, F.; Yang, J. Life Cycle Assessment of Lithium Nickel Cobalt Manganese Oxide (NCM) Batteries for Electric Passenger Vehicles. *J Clean Prod* **2020**, *273*. https://doi.org/10.1016/j.jclepro.2020.123006.

(22) Dai, Q.; Kelly, J. C.; Gaines, L.; Wang, M. Life Cycle Analysis of Lithium-Ion Batteries for Automotive Applications. *Batteries* **2019**, *5* (2), 48. https://doi.org/10.3390/batteries5020048.

(23) Peters, J. F.; Baumann, M.; Zimmermann, B.; Braun, J.; Weil, M. *The Environmental Impact of Li-Ion Batteries and the Role of Key Parameters – A Review*; Pergamon, 2017; Vol. 67, pp 491–506. https://doi.org/10.1016/j.rser.2016.08.039.

(24) Milovanoff, A.; Posen, I. D.; MacLean, H. L. Electrification of Light-Duty Vehicle Fleet Alone Will Not Meet Mitigation Targets. *Nat Clim Chang* **2020**, *10* (12), 1102–1107. https://doi.org/10.1038/s41558-020-00921-7.

(25) Majeau-Bettez, G.; Hawkins, T. R.; StrØmman, A. H. Life Cycle Environmental Assessment of Lithium-Ion and Nickel Metal Hydride Batteries for Plug-in Hybrid and Battery Electric Vehicles. *Environ Sci Technol* **2011**, *45* (10), 4548–4554. https://doi.org/10.1021/es103607c.

(26) Dai, Q.; Kelly, J. C.; Gaines, L.; Wang, M. Life Cycle Analysis of Lithium-Ion Batteries for Automotive Applications. *Batteries 2019, Vol. 5, Page 48* **2019**, *5* (2), 48. https://doi.org/10.3390/BATTERIES5020048.

(27) Ellingsen, L. A. W.; Hung, C. R.; Strømman, A. H. Identifying Key Assumptions and Differences in Life Cycle Assessment Studies of Lithium-Ion Traction Batteries with Focus on Greenhouse Gas Emissions. *Transp Res D Transp Environ* **2017**, *55*, 82–90. https://doi.org/10.1016/j.trd.2017.06.028.

(28) Onat, N. C.; Kucukvar, M.; Aboushaqrah, N. N. M.; Jabbar, R. How Sustainable Is Electric Mobility? A Comprehensive Sustainability Assessment Approach for the Case of Qatar. *Appl Energy* **2019**, *250*, 461–477. https://doi.org/10.1016/j.apenergy.2019.05.076.

(29) *Asian Batteries Power Global EV Fleet*. https://www.statista.com/chart/26584/worldwide-market-share-of-the-biggest-electric-vehicle-battery-makers/ (accessed 2022-03-31).

(30) International Energy Agency. *Data & Statistics - IEA*. Electricity Information. https://www.iea.org/data-and-statistics?country=WORLD&fuel=Energy supply&indicator=TPESbySource (accessed 2021-02-24).

(31) Cozzi, L.; Gould, T.; Bouckart, S.; Crow, D.; Kim, T.-Y.; McGlade, C.; Olejarnik, P.; Wanner, B.; Wetzel, D. World Energy Outlook 2020. **2020**, *2050* (October), 1–461.

(32) Schlömer S.; T. Bruckner, L.; Fulton A. McKinnon D. Perczyk J. Roy R. Schaeffer R. Sims P. Smith and R. Wiser, E. H. *Annex III: Technology-Specific Cost and Performance Parameters in Climate Change 2014:Mitigation of Climate Change. Contribution of Working Group III to the Fifth Assessment Report of the Intergovernmental Panel on Climate Change*; 2014.

(33) Tang, C.; Sprecher, B.; Tukker, A.; Mogollón, J. M. The Impact of Climate Policy Implementation on Lithium, Cobalt and Nickel Demand: The Case of the Dutch Automotive Sector up to 2040. *Resources Policy* **2021**, *74*, 102351. https://doi.org/10.1016/J.RESOURPOL.2021.102351.

(34) Usón, A. A.; Capilla, A. V.; Bribián, I. Z.; Scarpellini, S.; Sastresa, E. L. Energy Efficiency in Transport and Mobility from an Eco-Efficiency Viewpoint. *Energy* **2011**, *36* (4), 1916–1923. https://doi.org/10.1016/j.energy.2010.05.002.

(35) Sihvonen, J.; Mathieu, L. *Mission (Almost) Accomplished*; 2020.

(36) *Data and maps — European Environment Agency*. https://www.eea.europa.eu/data-and-maps (accessed 2021-02-24).

(37) Zhang, C.; Yang, F.; Ke, X.; Liu, Z.; Yuan, C. Predictive Modeling of Energy Consumption and Greenhouse Gas Emissions from Autonomous Electric Vehicle Operations. *Appl Energy* **2019**, *254*. https://doi.org/10.1016/j.apenergy.2019.113597.

(38) Cox, B.; Bauer, C.; Mendoza Beltran, A.; van Vuuren, D. P.; Mutel, C. L. Life Cycle Environmental and Cost Comparison of Current and Future Passenger Cars under Different Energy Scenarios. *Appl Energy* **2020**, *269*. https://doi.org/10.1016/j.apenergy.2020.115021.

(39) Milovanoff, A.; Kim, H. C.; De Kleine, R.; Wallington, T. J.; Daniel Posen, I.; Maclean, H. L. *Supporting Information: A Dynamic Fleet Model of U.S Light-Duty Vehicle Lightweighting and Associated Greenhouse Gas Emissions from 2016-2050*.
